# Water: Promising Opportunities For Tunable All-dielectric Electromagnetic Metamaterials

**DOI:** 10.1038/srep13535

**Published:** 2015-08-27

**Authors:** Andrei Andryieuski, Svetlana M. Kuznetsova, Sergei V. Zhukovsky, Yuri S. Kivshar, Andrei V. Lavrinenko

**Affiliations:** 1 DTU Fotonik, Technical University of Denmark, Ørsteds Plads 343, DK-2800 Kgs. Lyngby, Denmark; 2 Radiophysics Department, University of Nizhny Novgorod, Nizhny Novgorod 603950, Russia; 3 ITMO University, St. Petersburg 197101, Russia; 4 Nonlinear Physics Center, Australian National University, Canberra ACT 0200, Australia

## Abstract

We reveal an outstanding potential of water as an inexpensive, abundant and bio-friendly high-refractive-index material for creating tunable all-dielectric photonic structures and metamaterials. Specifically, we demonstrate thermal, mechanical and gravitational tunability of magnetic and electric resonances in a metamaterial consisting of periodically positioned water-filled reservoirs. The proposed water-based metamaterials can find applications not only as cheap and ecological microwave devices, but also in optical and terahertz metamaterials prototyping and educational lab equipment.

All-dielectric metamaterials^1^ are an attractive alternative to the resonant metal-based photonic structures due to smaller material losses. Being properly designed, such metamaterials can exhibit most of the properties of metamaterials: negative magnetic permeability^2^,^3^,^4^,^5^,^6^,^7^, negative^8^,^9^,^10^ and zero^11^ refractive index and even toroidal dipole moment^12^. Typically, all-dielectric metamaterials are composed of high-permittivity inclusions in a low dielectric matrix. The geometrical shape and dielectric permittivity *ε* of the inclusions are responsible for the extraordinary metamaterial properties^13^.

Relative electric permittivity *ε* of the inclusions typically does not exceed 50 in the optical and near-infrared ranges. As an example, silicon nanoparticles^14^,^15^, dimers^16^, nanorods^11^ and spheroids^17^ have recently been investigated. As the wavelengths get increased towards the micro- and radio-waves region, which is often used for faster and cheaper experimental prototyping of optical metamaterials and structures^18^,^19^, the range of available high-permittivity dielectrics is considerably broadened^20^, with permittivities of several hundreds becoming relatively common. “In a shadow” of these radio-frequency high-*ε* dielectrics (for example, barium strontium titanate), simpler materials, such as water with its relatively modest low-frequency Re(*ε*) ≈ 80 at room temperature, are often overlooked. In fact, water has not attracted any attention of the scientific community as a building block for electromagnetic metamaterials until very recently^21^, only being employed for negative-index acoustic metamaterials^22^,^23^ during the past few years.

Even though the real part of permittivity Re(*ε*) for water is relatively high (especially compared to the materials in the optical range), pure water is fairly lossy (the fact widely used for microwave cooking^24^), and the losses Im(*ε*) get further increased in presence of impurities ions. This has typically been viewed as a detriment to the use of water in a design of electromagnetic structures.

On the other hand, water is one of the most abundant, cheapest, and most bio-compatible materials on Earth, which is a tremendous advantage over scarce and expensive materials such as barium strontium titanate.

As is commonly known, water is a liquid in the temperature range from 0 to 100 °C at normal pressure. Therefore, it preserves its volume and takes the shape of its container, which opens up many possibilities for varying the shape of a water-based object using either mechanical deformation of an elastic container or gravity. Another prominent property of water is its dielectric permittivity temperature dependence (see Fig. 1), which is well described by the Debye formula^25^
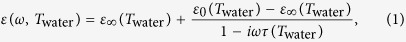
where *ε*_∞_ and *ε*_0_ are the optical and static permittivities respectively; *τ* is the rotational relaxation time (see Methods for more details). Such strong dependence enables the use of temperature as another mean to tune the electromagnetic properties of an aqueous object.

In this paper we demonstrate an outstanding potential of water as a building platform for inexpensive tunable all-dielectric electromagnetic metamaterials. We focus on thermal, mechanical and gravitational tunability of the metamaterial properties, and discuss further extensions of the proposed concept.

## Results

Throughout the paper we consider normal incidence of an electromagnetic wave on a single layer of meta-atoms made of water, i.e., water-filled thin-walled containers made of low-*ε* low-loss dielectric (for example, plastic or glass). Such meta-atoms are arranged in a square array with the sub-wavelength lattice constant *a* = 7.5 cm operating at frequencies *f* around 1 GHz to be in the metamaterial regime^26^ (frequency 1 GHz corresponds to wavelength *λ* = 30 cm and, thus, the metamaterial lattice constant *a* ≈ *λ*/4). The volume of water in each unit cell is 0.10*a*^3^ in all cases (i.e., the filling fraction of the water in all the considered metamaterials is fixed at 0.10). The meta-atoms are of various shapes, predominantly having axial symmetry (sphere, ellipsoid, elliptical cylinder etc.).

We consider the lowest-order metamaterials resonances, which are magnetic and electric dipoles. The properties of the meta-atoms can be well described with the normalized electric and magnetic polarizabilities:
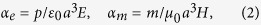
where *p* and *m* are electric and magnetic dipole moments of a single particle excited by electric *E* and magnetic *H* fields of an incident plane wave, respectively. Description of the homogenized metamaterials in terms of effective permittivity *ε*_eff_ and permeability *μ*_eff_ can then be related to the normalized polarizabilities of Eq. (2) together with the dipole interaction constant *β*, which depends on the meta-atoms spatial arrangement. In the considered case of the two-dimensional square lattice the interaction constant can be calculated analytically^27^
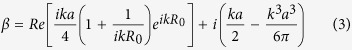
where *k* = 2*π*/*λ* is the wavenumber of an incident wave and *R*_0_ = *a*/1.438.

The amplitude reflection and transmission coefficients of the metamaterial slab, *r* and *t*, can therefore be related to the normalized polarizabilities of the meta-atoms (see Methods). After modelling or measuring *r* and *t* one can get the polarizabilities *α*_*e*_ and *α*_*m*_. The resulted expressions are^27^,^28^
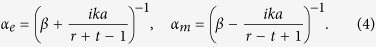


In our idealized model, we neglect the influence of the container material, as well as any supporting structures (holders, foam, ropes, sticks, etc.), on the metamaterial properties by assuming that the combination of low filling fraction and low *ε* in comparison with water makes the electromagnetic contribution of those auxiliary elements negligible.

### Thermal tunability

**Figure 1:**
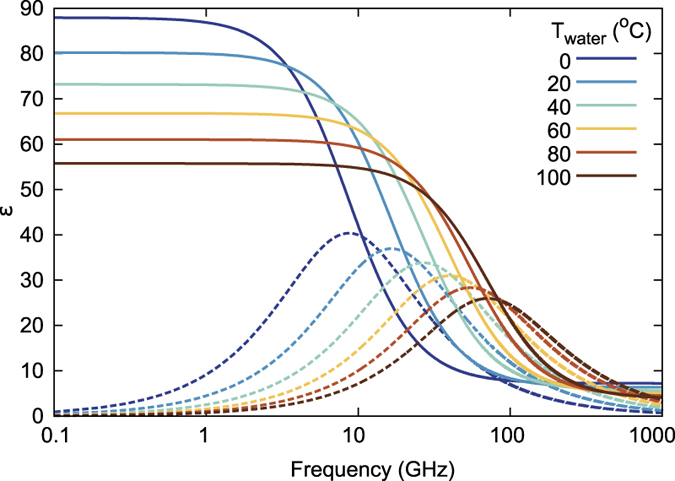
Dielectric permittivity of water as a function of frequency for the temperature 0–100 °C. Here and in further figures, solid lines correspond to the real part, dashed lines to the imaginary part.

**Figure 2:**
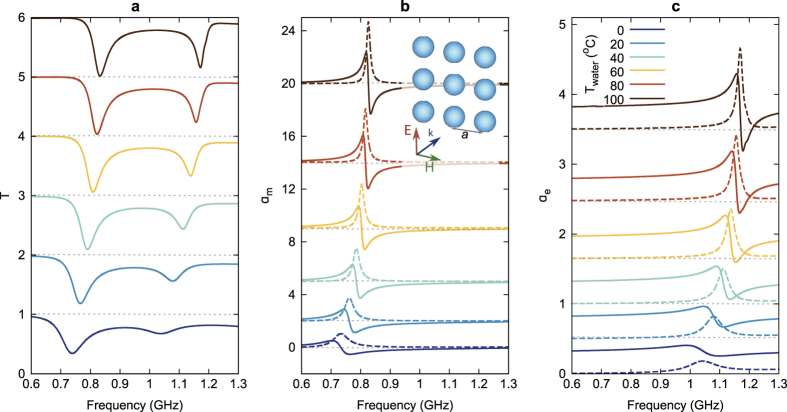
Thermal tunability of (a) transmittance, (b) magnetic and (c) electric polarizabilities of spherical particles array in the temperature range 0–100 °C. Solid and dashed lines correspond to the real and imaginary part of polarizabilities, respectively. The offset for each spectrum is shown as a dotted grey line. The inset shows the metasurface and the incident plane wave.

As discussed above, temperature increase from 0°C to 100 °C leads to water permittivity decrease from 86 to 50 (see Fig. 1). This has already been used for switching a water-filled glass cylinder from highly visible (by virtue of strong Mie scattering) to invisible^21^. Thus, temperature variation brings about the change of the resonant properties of water-filled containers, such as spheres of radius 2.16 cm in the example in Fig. 2. The transmittance of a square lattice of such spheres (Fig. 2a) exhibits two dips in the frequency range of interest. As one can infer from the retrieved magnetic (Fig. 2b) and electric (Fig. 2c) polarizabilities, the lower-frequency dip corresponds to a magnetic dipole resonance, while the higher-frequency dip corresponds to an electric dipole resonance; the same conclusion can be obtained by analysis of the field distributions (not shown). As temperature rises from 0 to 100 °C, the resonances experience the blue shift from 0.74 GHz to 0.83 GHz for the magnetic one and from 1.04 GHz to 1.17 GHz for the electric dipole one due to the decrease of Re(*ε*). Moreover, the resonances become narrower due to the falling down of Im(*ε*) of water with temperature increase in this frequency range.

**Figure 3:**
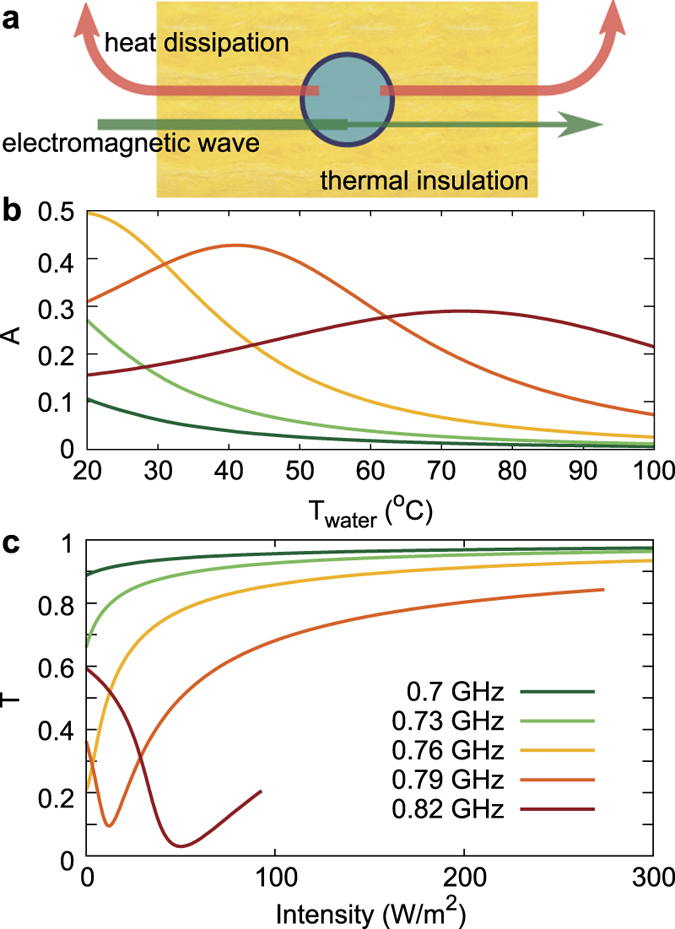
(a) Schematics of a meta-atom surrounded with a thermal insulation (30 cm of glass wool). The incident electromagnetic intensity is partially absorbed by the meta-atom. In the steady state the absorbed power converts into heat and then is released into the surrounding medium via heat conductance through the insulation and natural convection. (**b**) Absorbance as a function of water temperature and (**c**) steady-state transmittance as a function of the incident electromagnetic intensity at certain frequencies near the magnetic resonance. For 0.79 and 0.82 GHz, boiling point of water is reached at intensities below 300 W/m^2^.

The temperature of water *T*_water_ can be increased by various means, including the microwave radiation itself. This suggests a nonlinear regime of the water-based metamaterial, where the incident radiation heats the metamaterial and changes its properties, including absorbance *A* and transmittance *T*. Consider the layer of water-filled containers surrounded by thermal insulation (for example, 30 cm of glass wool on both sides) (Fig. 3a) at the frequencies around the magnetic resonance. Absorbance *A* at these frequencies (Fig. 3b) largely depends on *T*_water_, reaching a maximum value of 0.5 at *T*_water_ = 20 °C at *f* = 0.76 GHz. Note that 0.5 is maximal theoretically achievable absorbance for a thin metasurface with symmetric surroundings at the normal wave incidence conditions^29^.

In order to obtain the transmittance dependence on the incident power we solve the power balance equation *I*_0_*A*(*T*_water_) = *J*_*T*_ (*T*_water_), where *I*_0_ is the incident electromagnetic intensity and *J*_*T*_ (*T*_water_) is the heat dissipation power flux. The heat penetrates through the insulation and then dissipates through natural convection (see the calculation details in Methods). Solving the power balance equation we get water temperature *T*_water_(*I*_0_) and then transmittance *T*(*I*_0_) dependence on the incident intensity.

The resulting transmittance dependence on the incident intensity *T*(*I*_0_) is far from constant (Fig. 3c). For the frequency *f* = 0.76 GHz transmittance increases from 0.21 to 0.93 for the intensity change from 0 to 300 W/m^2^, while for the frequency *f* = 0.79 GHz it first drops from 0.36 to 0.10 and then grows to 0.84. Some of the graphs stop for the intensities below 300 W/m^2^ due to water reaching the boiling point. Reaching higher temperatures is also possible, though it requires the water containers to be firm enough to sustain higher pressure.

### Mechanical tunability

**Figure 4:**
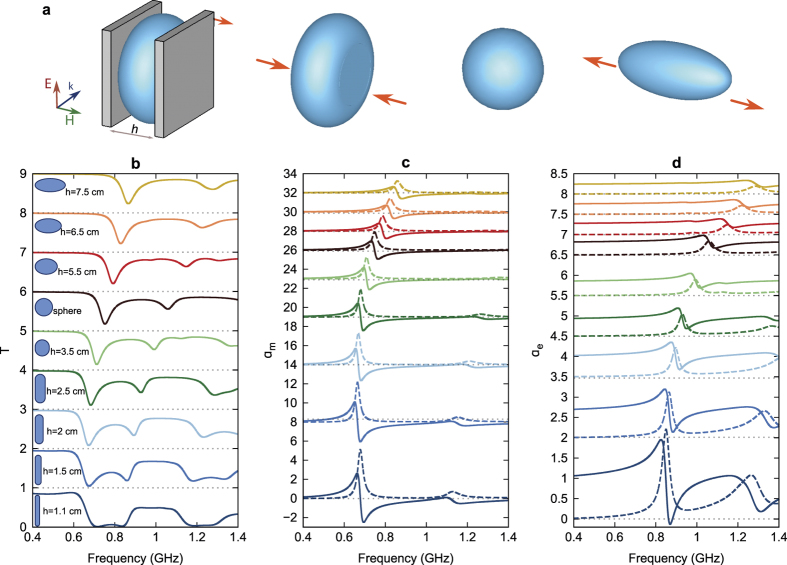
(a) Schematic view of the meta-atom undergoing elastic deformation processes. The elastic water-filled container is attached at two opposite points to the moving flat frames and undergoes shape transformation under compression (to a cylinder with toroidal edges) or stretching (to a prolate ellipsoid). (**b**) Transmittance spectra, (**c**) magnetic and (**d**) electric polarizabilities (real and imaginary parts shown by solid and dashed lines, respectively) for meta-atoms with different amount of applied compression and stretching along the *H*-field direction indicated on the graph.

Another important property of water is conservation of its volume. If water is placed into elastic containers (such as rubber balloons) subject to deformation, the resonant properties of such meta-atoms undergo significant changes. We consider the water-filled elastic spheres of the volume 0.10*a*^3^ fixed at two opposite points to a flat-surface moving frame (see Fig. 4a). Reducing the distance between the flat plates *h* and compressing the sphere makes it a cylinder with toroidal edges, while stretching the sphere results in a prolate ellipsoid. We neglect here the influence of the gravity on the flexible containers shape.

The transmittance spectra (Fig. 4b), magnetic (Fig. 4c) and electric (Fig. 4d) polarizabilities of the meta-atoms are changed with distance *h*, with resonance frequencies monotonously blue-shifting with *h* increase. As *h* varies from 1.1 cm (flattened cylinders) to 7.5 cm (stretched ellipsoids), the frequency of the electric resonance increases from 0.68 GHz to 0.86 GHz (Fig. 4c), while the frequency of the magnetic resonance increases from 0.83 GHz to 1.29 GHz (Fig. 4d).

### Gravitational tunability

**Figure 5:**
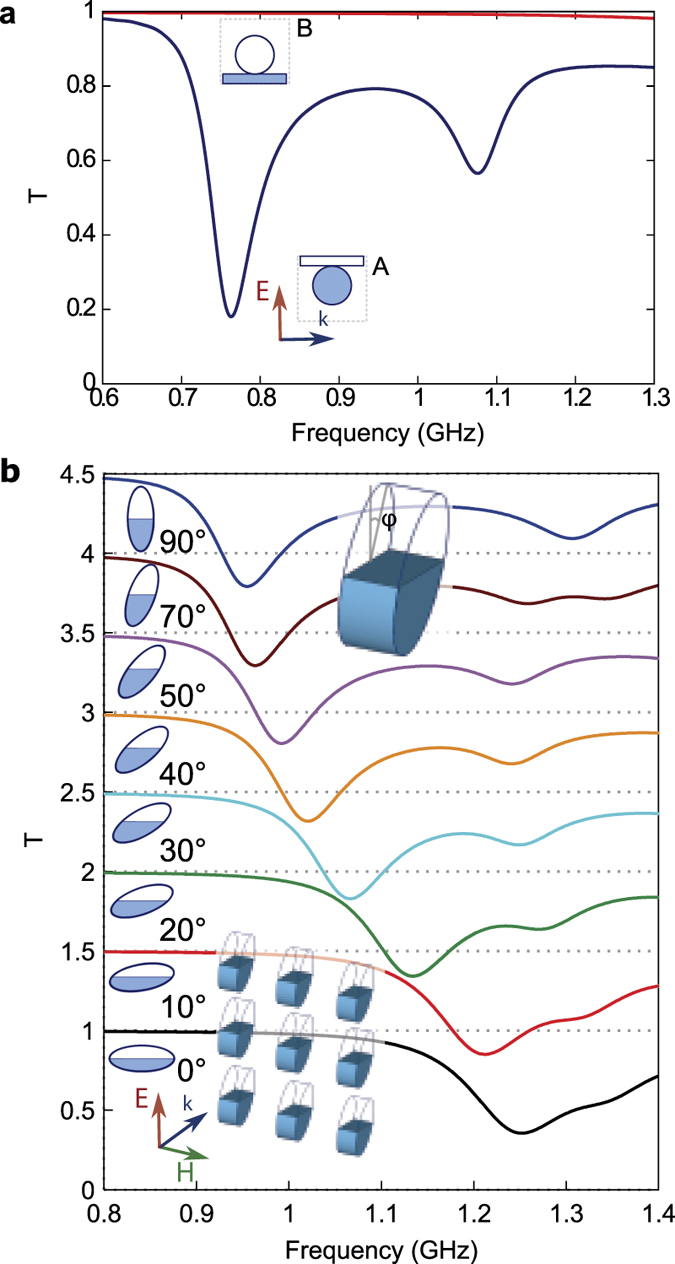
(a) Transmittance spectra of metamaterial consisting of connected containers of two kinds (spheres and square flat plates) exhibit two resonant dips in the position A (water filling the sphere), but switch to near unity transmittance for the position B (water fills the square plate). (**b**) Transmittance for partially filled rotating elliptical cylinders changes with its rotation around the direction of *H* due to gravity-induced water redistribution.

It is the well-known fact that water takes the shape of the provided container in such way that its potential energy (in a steady state) is minimized. In a homogeneous gravitational field such as exists on the Earth surface, this results in a flat surface of water perpendicular to the direction of gravity. This can be used as another way of metamaterial tunability and switching. Consider a unit cell consisting of two connected containers of different shape, but of the same volume, for instance, the previously considered sphere with radius 2.16 cm and a flat rectangular plate with sizes 7.0 × 7.0 × 0.86 cm^3^. Let only the sphere be initially filled with water (see inset A in Fig. 5a). The electromagnetic wave interacts with the metasurface as if it is an array of water-filled spheres, giving two dips in the transmittance spectra (Fig. 5a, case A) as described above. Turning the reservoirs upside down (Fig. 5a, case B) (e.g., by rotating the metasurface by 180 degrees) results in water redistribution to the flat plate, producing nearly unity resonance-free transmittance. This unit cell realizes a gravitational metamaterial transmittance switch (transmittance changes from 0.18 to 0.99 at frequency *f* = 0.76 GHz).

Instead of two reservoirs with different shapes (the switching scenario), one may employ a partially filled single reservoir with a certain degree of asymmetry, such as an elliptical cylinder. When such elliptical cylinders are rotated, the water is redistributed due to gravity, so the effective shape of the meta-atom is varied, which in turn gradually changes the metamaterial properties (the tuning scenario).

As an example, we consider an elliptical cylinder with major axis 7.5 cm, minor axis 3.75 cm and thickness 1.91 cm, half-filled with water and rotating around the *H*—field direction (see Fig. 5b). The transmission dip corresponding to the magnetic resonance gradually shifts from 1.25 GHz to 0.97 GHz with the rotation angle *ϕ* varied from 0 to 90 degrees. The shift of the other peak (corresponding to the electric resonance) is non-monotonous. Strictly speaking, any orientation except 0° and 90° angle makes the meta atoms asymmetric along the wave propagation direction, so the metamaterial becomes bianisotropic^30^. Thus the electric and magnetic dipole polarizabilities are no longer sufficient to characterize its properties and an additional magneto-electric coupling should be taken into account.

## Discussion

We have demonstrated that water can be used as a very cheap and versatile building block for electromagnetic metamaterials, using several options for thermal, mechanical and gravitational tuning. However, these three possibilities, along with the accompanying numerical demonstrations, are just to exemplify the possible versatility of water-based metamaterials, and there are much broader opportunities to affect their electromagnetic properties.

As we have shown, heating causes not only resonance shift, but also change of the water material loss and thus modification of the resonator quality factor. Heating may be provided with a multitude of options, including light, microwaves, chemical reactions, radioactivity, mechanical friction and passing electrical current through resistors. In the latter case the metallic wires supplying current to the miniature heaters can be arranged in a periodic way and thus comprise a broadband negative permittivity wire medium^31^.Water-based meta-atoms at the magnetic resonance together with a wire medium would possibly provide a tunable negative index metamaterial.

Moreover, cooling of water below 0 °C turns it into ice with a dramatic change of permittivity from 86 to 3 upon this phase transition^32^; this transition would switch off all the resonances related to the high Re(*ε*) of liquid water.

We have also shown the thermal nonlinear regime appearing from water meta-atoms heated by the energy of the incident microwave. We would like to highlight the fact that the nonlinear regime does not require large electromagnetic intensities, as small as 100 W/m^2^ are enough, such intensities can be easily achieved even with a household microwave oven. Nonlinearity can be controlled by thermal insulation (for example, cheap glass wool widely used in construction). Moreover, a metallic mirror or an additional metamaterial layer can be used in order to increase the metamaterial absorbance from the single-surface theoretical maximum of 0.5 to unity. More sophisticated temperature control systems may allow not only for precise regulation of the metamaterial properties, but also for gradual parameters spatial distribution allowing for even more complex functional devices such as metamaterials holograms, flat lenses or carpet cloaks.

Mechanical motion and gravitation allow for tuning due to water redistribution. We have demonstrated tunability with rather simple compression/stretching of elastic reservoirs and rotation in the constant gravitational field. More complex configurations are possible, including active pumps to fill the containers with water, active dynamic structures such as periodic and aperiodic water jets and droplets, as well as air bubbles in water, along with the use of mechanical vibrations or ultrasound.

Gravitation is nearly homogeneous on the Earth surface, at least on the scale of the possible metamaterial device (up to a few meters). There are much richer options of “effective gravity” with the help of inertia forces due to linear motion or rotation. For example, water in partially filled containers on a rotating plate can be distributed differently depending on the distance to the rotation axis and angular velocity. This allows for constructing a metamaterial lens with a focal distance controlled by the rotation speed.

Similar concepts can be applied to other materials in the liquid state, including room-temperature liquid metals, high-index liquids or, in principle, to any melted materials. The latter option can be especially interesting for high-temperature metamaterials. Moreover, the metamaterials can be scaled up or down to other frequency range.

We admit that water based metamaterials cannot compete with other switchable or tunable microwave devices in terms of speed. Indeed, metamaterials with integrated field-effect transistors can operate with the switching speed of more than 10 MHz^33^ and potentially up to several hundreds of GHz with graphene based transistors^34^, while water metamaterials are limited by slow thermal or water redistribution processes and can hardly be faster than a few Hz. However, the main advantage of water is its extremely low cost, abundance and ecological compatibility. Various methods can be used for water metamaterials fabrication starting from the simplest ones like water-filling ice cube bags to more complicated ones requiring plastic forms moulding or 3D additive manufacturing. The suggested metamaterials can be created literally from trash (plastic bottles, rubber balloons, plastic bags etc.) Moreover, the sources of the microwave radiation are also inexpensive. This provides not only a way of quick and cheap prototyping of metamaterials, but also their wide applications for educational and science popularization purposes, including extremely low-budget options readily affordable by the developing world.

## Methods

### Water permittivity

The quantities appearing in the Debye formula are modelled as following^25^:
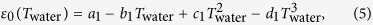

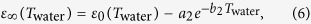

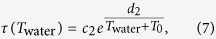
where *a*_1_ = 87.9, *b*_1_ = 0.404 *K*^−1^, *c*_1_ = 9.59 × 10^−4^ *K*^−2^, *d*_1_ = 1.33 × 10^−6^ *K*^−3^, *a*_2_ = 80.7, *b*_2_ = 4.42 × 10^−3^*K*^−1^, *c*_2_ = 1.37 × 10^−13^ *s, d*_2_ = 651 °C, *T*_0_ = 133 °C and *T*_water_ is the water temperature in °C.

### Electric and magnetic polarizabilities retrieval

Every meta-atom is considered as a conjunction of point electric and magnetic dipoles, excited by an incident plane wave with no cross-coupling between electric and magnetic dipole moments. The dipole moments of a reference meta-atom are related to the incident field *E*_0_, *H*_0_ and to the fields of all the neighbours *E*_*inter*_, *H*_*inter*_ via
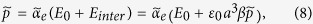

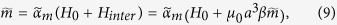
where *β* is the interaction constant and 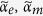 are the electric and magnetic polarizabilities of meta-atoms. The reflection and transmission coefficients, which can be divided into electric and magnetic parts, are expressed in terms of polarizabilities^35^:
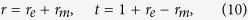

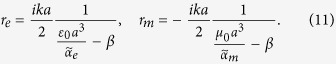


The normalized polarizabilities of the meta-atoms are then extracted from the reflection data:
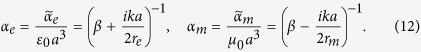

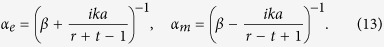


### Numerical simulation

Numerical simulations were done with CST Microwave Studio^36^ in the time-domain with a hexahedral (rectangular) fine mesh and effectively periodic (x-perfect electric, y-perfect magnetic) boundary conditions.

### Power balance equation

In order to obtain the dependence of the transmittance on the incident intensity, we cover the metasurface with thermal insulator from both sides. To simplify the heat transfer problem we substitute the spherical meta-atoms with a flat layer of temperature *T*_water_ with the thickness equal to the spheres diameter. In a steady state the dissipated power flux is expressed as
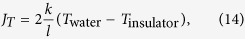
where the factor 2 stands for two insulation sides (left and right), *l* is the thickness of the insulator layer, *k* is the thermal conductivity of insulator and *T*_insulator_ is the temperature of the insulator outer surface. We considered the glass wool of 30 cm from each side of the array as an insulating material and in the temperature range of interest *k* was considered constant and equal^37^ to 0.04 W/(K · m).

In the thermal equilibrium the flux through the insulation should be equal to the flux dissipated with natural convection which takes place on the outer surface of the insulator^38^:
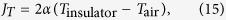
where *α* is the heat transfer coefficient of air and is related to Nusselt number *Nu* and air thermal conductivity *k*_air_ via *α* = *Nu* · *k*_air_/*L* (*L* is a dimension of a metasurface, along which convection takes place. We assumed the metamaterial to be a square array of the size *L* = 2.1 m). In general case *Nu* = *C*(*Ra*)^*n*^, where *C*,*n* are constants, which depend on the air motion regime and *Ra* = *Pr* · *Gr* is the Rayleigh number, which is a product of Prandtl number *Pr* and Grashof number *Gr*:
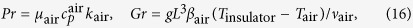
where *μ*_air_ is the air dynamic viscosity, 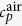 is its specific heat capacity, *g* is the gravity, *β*_air_ is the air temperature coefficient of volume expansion and *v*_air_ is the air kinematic viscosity. Since the properties of gases depend on their temperature, *v*_air_, *μ*_air_, 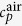 and *k*_air_ should be taken at a characteristic absolute temperature 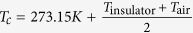. In the turbulent regime (10^9^ < *Ra* < 10^13^) *C* = 0.1, *n* = 1/3 and in the laminar regime (*Ra* < 10^9^) *C* = 0.59, n = 1/4^38^,^39^. The above mentioned parameters may be interpolated as^38^
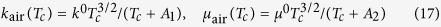
where *k*^0^ = 0.254 × 10^−2^ W/(K · m), *μ*^0^ = 1.46 × 10^−6^ Ns/m^2^, *A*_1_ = 201 K, *A*_2_ = 110.4 K.

From equations (14) and (15) one obtains
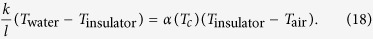


Solving it we get the outer insulator temperature dependence on the water temperature *T*_surface_ (*T*_water_) and then can obtain the total dissipated power flux
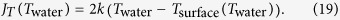


Dependence of *J*_*T*_ (*T*_water_) is close to linear 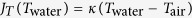 with the coefficient 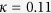
W/m^2^K.

In the steady regime the thermal power flux equals to the intensity absorbed by water *A*(*T*_water_)*I*_0_. This allows one to determine the temperature of water, solving the equation
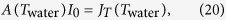
and relate transmittance T to incident intensity *I*_0_ as *T* (*I*_0_) = *T* (*T*_water_(*I*_0_)).

## Additional Information

**How to cite this article**: Andryieuski, A. *et al.* Water: Promising Opportunities For Tunable All-dielectric Electromagnetic Metamaterials. *Sci. Rep.* **5**, 13535; doi: 10.1038/srep13535 (2015).
